# Commentary: Environmental Sound Training in Cochlear Implant Users

**DOI:** 10.3389/fnins.2017.00036

**Published:** 2017-02-01

**Authors:** Nicholas Altieri

**Affiliations:** Communication Sciences and Disorders, ISU Multimodal Language Processing Lab, Idaho State UniversityPocatello, ID, USA

**Keywords:** cochlear implants, multimodal perception, environmental sound training, individual differences, generalization

## Introduction

Cochlear implants (CIs) are prosthetic devices developed for listeners with profound bilateral hearing loss. Despite considerable advances in CI hearing technology allowing for improved speech and language recognition, several studies have reported that the identification of common environmental sounds—even years after implantation plus high speech perception scores—prove difficult for most listeners (e.g., Loebach and Pisoni, 2008; Shafiro et al., 2011, 2015). The literature suggests explanations for environmental sound identification difficulty in CI users: The chief difficulty is that CI signals are highly degraded compared to the frequency-rich neural signal in normal-hearing (NH) listeners. CIs typically include 4 to 22 electrodes; this electrode array, while constituting a drastic improvement from early CIs containing 1 to 4 electrodes, still represents less than 1% of hair cells in a healthy cochlea contributing to sound-frequency information (Wilson, 2004). Besides the degraded signal provided by even state-of-the-art CIs, Shafiro et al. (2015) described other factors complicating environmental sound identification, namely, the likelihood of degraded representations of memory for environmental sounds caused by years of hearing loss.

To help address these concerns, I propose a modification of the environmental sound training procedure initially developed by Shafiro et al. (2015). The aim is to utilize multisensory cues, sounds presented in noise to enhance ecological validity, and a same-different discrimination phase prior to closed-set identification. This modified procedure should enhance neural plasticity, and consequently reconstruct auditory representations that have become degraded after years of CI use.

## Training program overview

Shafiro et al. (2015) reviewed studies utilizing training programs involving presenting post-lingually deafened CI users with environmental sounds (e.g., Inverso and Limb, 2010; Looi and Arnephy, 2010), or alternatively, presenting NH listeners with either 4 or 8-channel simulated CI signals (Loebach and Pisoni, 2008; Shafiro et al., 2012). Results consistently showed evidence for significant improvement in listeners' ability to identify environmental sounds subsequent to closed-set training. Interestingly, evidence for generalization to other categories was reported, including improved scores in speech recognition by Loebach and Pisoni (2008) and Shafiro et al. (2012) (who examined simulated sounds in NH listeners).

In light of this research showing evidence for improved sound identification, Shafiro et al. (2015) developed a program to train post-lingually deafened CI users on a large closed-set of common sounds, and provide of a short 1-week computerized training program. The procedure consisted of two Pre-Test sessions separated by a week, another week of Training, and two Post-Test sessions each separated by 1 week. Each of these four sessions included two speech recognition tests (the CNC word recognition test; Peterson and Lehiste, 1962, and speech-in-noise SPIN-R; Elliott, 1995). Additionally, the Familiar Environmental Sound Test (FEST) was administered (Shafiro, 2008); FEST includes closed-set identification of 60 familiar sounds (160 words total; four tokens each) across five categories.

Sound-training involved training listeners on a subset of sounds obtained from FEST. On each training trial, a sound was presented and the listener was required to make a closed-set identification response. Feedback was critical to training: When a listener responded incorrectly, the program repeated the correct response three times before advancing to the next trial.

Shafiro et al.'s (2015) results indicated improved performance. Trained items showed the largest degree of improvement. Generalization was reported for untrained items, although performance on untrained items was substantially lower. Generalization, however, failed to occur for word or sentence recognition. Significant individual variability in environmental sound recognition skills was reported subsequent to training. Unfortunately, the authors observed that neither CI brand, length of implantation, nor age accounted for the variability. Variability was also observed across stimuli, with five items receiving particularly low identification scores even after training (e.g., “brushing teeth,” “blowing nose,” “zipper,” and “airplane flying”). Such sounds are “inharmonic,” possessing unique envelope cues that prove difficult for CI users to access.

## Optimizing environmental sound-training

To remedy these concerns, I propose a modified multimodal training procedure designed to improve sound-cue acquisition in CI users. Importantly, Shafiro et al. (2015) training utilized feedback. Incorrect responses were repeated three times before continuing. The first proposed modification will involve hierarchically structuring feedback: Each time a listener responds incorrectly, the first cue reinforcement will be to present the (without noise) with a video clip of the sound source. Next, the video will be removed and the same sound (or another token of the same sound) will be presented (again, without noise). The third cue will simply be a presentation of the sound at the same level of background noise used in testing. Studies on a wide variety of topics, from stroke patients with aphasia to traumatic brain injury patient with cognitive deficits support the efficacy of hierarchical cueing (Constantinidou et al., 2008; Abel et al., 2015). In an fMRI study examining the influence of hierarchical cueing therapy on brain reorganization in aphasia patients, Abel et al. (2015) reported that therapy gains appeared were associated with a decrease in brain activation. The observed activation decrease in the experimental group suggests that therapy gains facilitated *efficient* brain reorganization; efficient in the sense that less brain activation was required to perform the task.

Next, I suggest modifying the procedure by including a same-different detection phase (Phase 1) to reinforce and help encode representations (using two tokens on *same* trials)—this is especially important for difficult sounds such as “zippers.” Distinguishing “same” vs. “different” requires a lower-level cognitive decision; the ability to distinguish “same” vs. “different” is necessary although not sufficient for identification. Phase 2 will include the identification phase used by Shafiro albeit with the modified cueing procedure (Table 1). In controlled studies, these modifications will hypothetically reinforce auditory representations, improve generalization scores, and reduce variability among listeners and stimulus items.

**Table 1:** **Comparison of proposed training procedures**.

**Shafiro's training program**	**Proposed modification**
Include Multiple Tokens of Sounds	Include Multiple Tokens of Sounds
Closed-Set Identification	Phase 1: Same-Different Discrimination
Stimuli presented in quiet	Stimuli presented in noise to improve ecological validity
Feedback for incorrect responses: Repeat stimulus three times	Feedback for incorrect responses: Step 1—present sound with video Step 2—present sound in quiet Step 3—present sound in noise
Only one phase	Phase 2: Closed-Set Identification
	Stimuli presented in noise to improve ecological validity
	Feedback for incorrect responses: Step 1—present sound with video Step 2—present sound in quiet Step 3—present sound in noise

## Author contributions

The author confirms being the sole contributor of this work and approved it for publication.

### Conflict of interest statement

The author declares that the research was conducted in the absence of any commercial or financial relationships that could be construed as a potential conflict of interest.
